# Haemoglobin C and S Role in Acquired Immunity against *Plasmodium falciparum* Malaria

**DOI:** 10.1371/journal.pone.0000978

**Published:** 2007-10-03

**Authors:** Federica Verra, Jacques Simpore, George M. Warimwe, Kevin K. Tetteh, Tevis Howard, Faith H. A. Osier, Germana Bancone, Pamela Avellino, Isa Blot, Greg Fegan, Peter C. Bull, Thomas N. Williams, David J. Conway, Kevin Marsh, David Modiano

**Affiliations:** 1 Dipartimento di Scienze di Sanità Pubblica, Sezione di Parassitologia, University of Rome-La Sapienza, Rome, Italy; 2 Department of Infectious and Tropical Diseases, London School of Hygiene and Tropical Medicine, London, United Kingdom; 3 Centre Medical Saint Camille, Ouagadougou, Burkina Faso; 4 Kenya Medical Research Institute, Wellcome Trust Programme, Centre for Geographic Medicine Research, Kilifi District Hospital, Kilifi, Kenya; 5 Centre National de Transfusion Sanguine, Ouagadougou, Burkina Faso; 6 Medical Research Council Laboratories, Fajara, Banjul, The Gambia; University of Liverpool, United Kingdom

## Abstract

A recently proposed mechanism of protection for haemoglobin C (HbC; β6Glu→Lys) links an abnormal display of *Pf*EMP1, an antigen involved in malaria pathogenesis, on the surface of HbC infected erythrocytes together with the observation of reduced cytoadhesion of parasitized erythrocytes and impaired rosetting *in vitro*. We investigated the impact of this hypothesis on the development of acquired immunity against *Plasmodium falciparum* variant surface antigens (VSA) encoding *Pf*EMP1 in HbC in comparison with HbA and HbS carriers of Burkina Faso. We measured: *i*) total IgG against a single VSA, A4U, and against a panel of VSA from severe malaria cases in human sera from urban and rural areas of Burkina Faso of different haemoglobin genotypes (CC, AC, AS, SC, SS); *ii*) total IgG against recombinant proteins of *P. falciparum* asexual sporozoite, blood stage antigens, and parasite schizont extract; *iii*) total IgG against tetanus toxoid. Results showed that the reported abnormal cell-surface display of *Pf*EMP1 on HbC infected erythrocytes observed *in vitro* is not associated to lower anti- *Pf*EMP1 response *in vivo*. Higher immune response against the VSA panel and malaria antigens were observed in all adaptive genotypes containing at least one allelic variant HbC or HbS in the low transmission urban area whereas no differences were detected in the high transmission rural area. In both contexts the response against tetanus toxoid was not influenced by the β-globin genotype. These findings suggest that both HbC and HbS affect the early development of naturally acquired immunity against malaria. The enhanced immune reactivity in both HbC and HbS carriers supports the hypothesis that the protection against malaria of these adaptive genotypes might be at least partially mediated by acquired immunity against malaria.

## Introduction

Red blood cell disorders including haemoglobin variants and thalassaemias provide with their unusually high prevalence and distribution in malaria endemic areas [1]–[3] the most compelling evidence of genetic factors controlling the susceptibility to any infectious disease in humans. Conclusive evidence exists on the protective role of Haemoglobin C (HbC; β6Glu→Lys) against clinical *Plasmodium falciparum* malaria [4]–[6]. Recently, an abnormal display of *Pf*EMP1, an antigen involved in malaria pathogenesis, was reported [7], [8] on HbAC and HbCC infected erythrocytes that showed reduced cytoadhesion and impaired rosetting *in vitro*. On this basis it has been proposed that HbC protection might be attributed to the reduced *Pf*EMP1-mediated adherence of parasitized erythrocytes in the microvasculature. Given the reported observations, it could be hypothesized that HbC carriers may develop an altered immune response to *Pf*EMP1. Intriguingly, an enhanced immune recognition of variant surface antigens (VSA) was initially observed in HbAS individuals by Marsh et al., [9] and further demonstrated in a study showing that the presence of HbAS genotype was associated with enhanced recognition of two randomly selected clinical isolates amongst Gabonese children [10]. A cohort study in Kenya lends further support to the hypothesis of an accelerated acquisition of immunity against mild clinical malaria in HbAS children <10 years [11].

The coexistence of HbC and HbS in a hyperendemic malaria context such as Burkina Faso provided a unique opportunity to explore this question in a comparative approach. In the present study, we compared the humoral response to *Pf*EMP1 in individuals belonging to the Mossi ethnic group carrying different β-globin genotypes (AA, AC, CC, AS, SC, SS) living in urban and rural areas of Burkina Faso, West Africa by measuring: *i*) total IgG against a single VSA (variant surface antigen), A4U, and against a panel of VSA from severe malaria cases; *ii*) total IgG against recombinant proteins of *P. falciparum* sporozoite (CSP), blood stage (AMA1, EBA-175, MSP-1_19_, MSP-2, MSP-3) antigens, parasite schizont extract, and *iii*) total IgG against a non malaria antigen, tetanus toxoid.

## Materials and Methods

### Study population

Healthy individuals belonging to the Mossi ethnic group of Burkina Faso were recruited after oral informed consent was obtained during the dry seasons 2005/2006 at the “Centre Medical Saint Camille” of the capital city Ouagadougou, and in the villages nearby (Donsin, Kuiti, Nagbagre') of the Kadiogo district, as described in Table 1, within a survey of haemoglobinopathies approved by the Ethics Committee of the Saint Camille Medical Center according to the guidelines of the Ministry of Health of Burkina Faso. Haemoglobin genotype was determined by cellulose acetate electrophoresis (Helena). Parasite genomic DNA was extracted using QIAamp DNA Mini Kit (Qiagen). *Plasmodium falciparum* genotyping of *glurp* was carried out according to a standard protocol [12] to evaluate parasite positivity assessed by PCR at the time of sera collection.

**Table 1:** Study samples of Mossi ethnic group of Burkina Faso.

Geographic Origin	Period of blood collection	Haemoglobin genotype						
Rural samples (EIR>100)		AA	AC	CC	AS	SC	SS	Total
Donsin	Dry season (April 2006 )	46 (16.0)	16 (11.7)	4 (9.0)	2 (8.5)	2 (13.0)	-	70
Kuiti	Dry season (April 2006 )	20 (10.7)	11 (10.2)	8 (9.2)	5 (10.2)	2 (9.0)	2 (9.5)	48
Nagbagré	Dry season (April 2006 )	-	-	2+1* (15.0)	-	-	-	3
Urban samples (EIR:1-10)								
Ouagadougou	Dry season (January–May 2005)	-	80 (30.0)**	10 (27.4)***	40 (29.5)§	8 (19.2)§§	2 (8.0)	140
Ouagadougou	Dry season (January–May 2006)	86 (29.0)&	26 (24.5)&&	3	8 (32.5)&&&	3 (18.3)	-	126
Total		152	133	28	55	15	4	387

Individuals are indicated for each genotype and origin; age is given in brackets.

* recruited in December 2004;

** age available for 50 subjects;

*** age available only for 5 subjects;

§ age available only for 29 subjects;

§§ age available only for 6 subjects;

& age available only for 63 subjects;

&& age available only for 22 subjects;

&&& age available only for 6 subjects.

### Antibody Assays

Antibody responses against the parasite infected-erythrocyte surface antigens were tested using the method with modifications of Williams et al. [13] on 85 urban and 85 rural individuals including all haemoglobin genotypes which were processed in a single assay against *i*) a reference isolate A4U, derived by sequential selection for binding to the monoclonal antibody BC6, which is specific to the expressed A4U *var* gene, resulting in a population of infected erythrocytes expressing predominantly one specific *Pf*EMP1 variant on their surface, A4U *Pf*EMP1 [14] and *ii*) a composite isolate (CI) derived by pooling a panel of wild isolates obtained from children presenting to the wards of Kilifi district hospital in Kenya with malaria at high parasitemia (T.H and T.N.W unpublished data).

#### FACS analysis

Mature trophozoite stage parasitized RBC (pRBC) at between 3–5% parasitaemia were thawed from frozen culture using saline solutions according to a gradient from 12% to 0.9% in a suspension at 50% haematocrit with buffer (0.5% BSA/PBS). 1 µl of serum was pipetted into separate wells of a 96-well U-bottomed plate (Falcon, Becton Dickinson, USA) to which was added 11.5 µl of the infected pRBC cell suspension in 0.5%BSA/PBS and 10 µg/ml of ethidium bromide. The mixture was incubated at room temperature for 30 min, following which the cells were washed three times with 0.5%BSA/PBS, centrifuging at 1000 rpm for 3 min per wash. The cells were then re-suspended in 50 µl 0.5%BSA/PBS containing a 1:50 dilution of sheep anti-human γ chain (FITC) fluorescein isothiocyanate-conjugated antibody (The Binding Site) was added to each well. A further incubation at room temperature in darkness, for 30 min was carried out, after which, following a further series of washes, at least 1000 pRBC were counted on an EPIC/XL flow cytometer (Coulter, UK). The Mean Fluorescence Intensity (MFI) was defined as the difference between the geometric mean of the fluorescence emitted by trophozoite pRBC and the geometric mean of the fluorescence emitted by the non pRBC. Non-specific recognition as measured by European negative controls was subtracted by the MFI of tested individuals.

#### ELISA analysis

Enzyme linked immunosorbant assays (ELISA) were performed according to well established protocols [15], [16]. Assays were performed in duplicate for each serum sample (N = 387) against the circumsporozoite protein (CSP) of *P. falciparum* (NANP_16_ CDC reagent gift from Patrick Corran, LSHTM, London, UK) and several blood stage antigens: full length 3D7 allele of AMA-1 (gift from David Lanar, Walter Reed Army Institute for Research, Washington, DC), Wellcome allele of MSP-1_19_ (gift from Patrick Corran, LSHTM, with permission of Tony Holder, London, UK), Dd2 allele of MSP-2 (gift from David Cavanagh, Institute of Immunology and Infection Research, Edinburgh, UK), 3D7 allele of MSP-3 (gift from Spencer Polley, LSHTM, London, UK), Camp allele F2 domain of EBA-175 region II (gift from Chetan Chitnis, ICGEB, New Delhi, India), and the Wellcome strain parasite schizont extract (gift from David Walliker, Institute of Cell, Animal and Population Biology, Edinburgh prepared by Lindsay Stewart, LSTHM, London, UK). When antigens were conjugated to GST or MBP, the optical density (OD) of GST or MBP for each sample was subtracted from that of the antigen to obtain the final OD. All assays were performed in Dynex Immunolon 4HBX ELISA plates (Dynex Technologies Inc). Wells were coated with 50 ng for all blood stage antigens (1 µg for CSP and PSE) in 100 µl of carbonate buffer (15 mM Na_2_CO_3_, 35 mM NAHCO3, pH 9.3), and incubated overnight at 4°C before washing four times in PBS/Tween (Phosphate Buffered Saline/0.05% Tween 20). The plates were then incubated in 200 µl/well of blocking buffer (1% skimmed milk in PBS/Tween) for 5 hours at room temperature. Antigen-coated wells were washed and incubated overnight at 4°C with test sera (1/500 dilution) at 100 µl/well. Unbound antibody was washed off, and 100 µl of secondary antibody (HRP-conjugated rabbit anti-human IgG, Dako Ltd.) diluted to 1/5000 in blocking buffer was added into each well and incubated for 3 hours at room temperature before detection with O-phenylenediamine (OPD, Sigma). The reaction was stopped with 25 µl/well of 2M H_2_SO_4_. The absorbance was read at OD 492nm using the SPECTRAFluor program, (XFLUOR4 Version: V 4.11) and analysed using Excel (Microsoft). The coefficient of variation (CV) between duplicate wells was calculated in Excel (Microsoft) using the formula below to ensure accuracy: 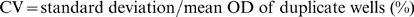 The mean OD of each duplicate was used as the final read-out. The cut-off for positive samples was determined by taking the mean plus 3 standard deviations of twenty (20) non-immune sera for all antigens. All samples were tested for each antigen and its conjugated molecule in a single assay to avoid any daily variation. OD values were log-transformed for normal distribution and differences in means of antibody levels and MFIs according to the different genotypes were tested by Kruskal-Wallis and pairwise comparisons by Two Sample T test. For all tests, P values of less than 0.05 were considered significant. Logistic regression was used to examine the relationship between antibody levels and the chosen variables: Hb genotype, age categories (1/9, 10/max years), gender and parasite positivity assessed by PCR. All analyses were carried out in STATA (StataCorp.1999, Release (9.2)).

## Results

### Serological reactivities to *P. falciparum* VSA

**Figure 1:**
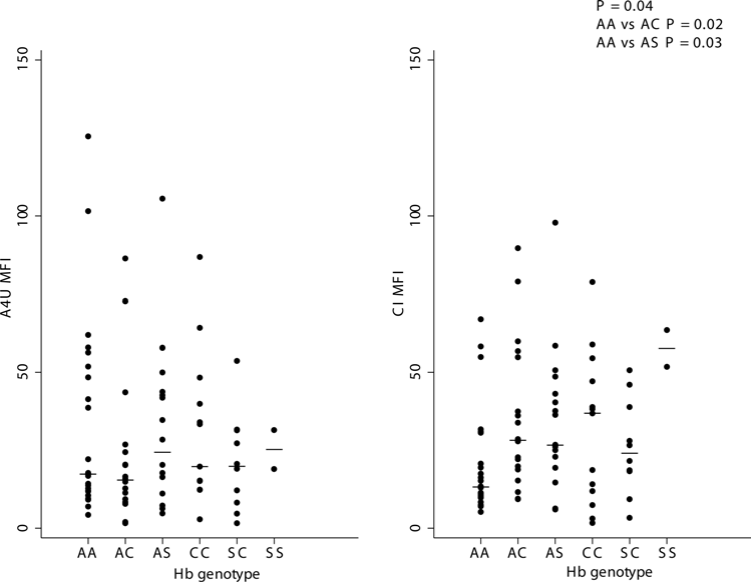
Mean Fluorescence Intensity (MFI) of total IgG against *Plasmodium falciparum* A4 Ultra parasite isolate (A4U) and a panel of Composite Isolates (CI) expressing VSA in urban samples of Mossi from Ouagadougou, Burkina Faso according to the different haemoglobin genotypes.

No differences in the Mean Fluorescence Intensity (MFI) according to the haemoglobin genotype were detected when looking at urban and rural samples all together. Further comparisons were carried out separately with the urban and the rural samples due to the different EIR (entomological inoculation rate) and mean age of the two subsets whose characteristics are described in Table 1. No differences were observed in the MFI of the different haemoglobin genotypes against the A4U isolate in both subsets, whereas we found significant differences in the urban sera (P = 0.04 amongst all adaptive genotypes and HbAA; P = 0.02 between HbAA and HbAC; P = 0.03 between HbAA and HbAS) when testing the panel of composite isolates (CI) of severe malaria VSA from Kilifi (Figure 1).

### Serological reactivities to *P. falciparum* antigens and parasite schizont extract

**Figure 2:**
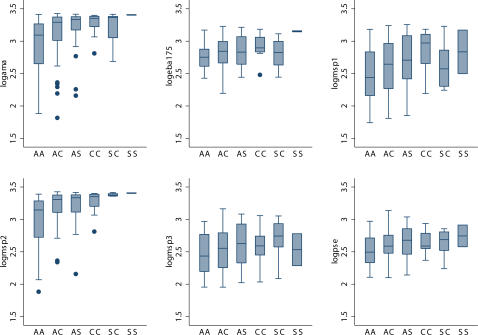
Means levels of total IgG against several blood stage antigens of *Plasmodium falciparum* (AMA1, EBA-175, MSP-1_19_, MSP-2, MSP-3) and parasite schizont extract in urban samples of Mossi from Ouagadougou, Burkina Faso according to the different haemoglobin genotypes. The horizontal lines in each box correspond to the median values, the lower edge of each box is the 25% ile and the upper edge is the 75% ile. The whiskers represent the range of the data beyond these percentiles, excluding outliers represented by dots.

Prevalence and levels of antibodies tested against all antigens was consistently higher in the villages due to higher exposure to malaria of the rural subset despite the relatively younger age (Table 2, Table 3). Within the urban samples higher levels of total IgG amongst genotypes containing at least one adaptive haemoglobin allele compared to HbAA were observed for most of the *P. falciparum* antigens tested by ELISA, with some degree of statistical significance as measured by Kruskal-Wallis test and pairwise T-test (Table 2, Figure 2). Remarkably, the same observation of higher levels of IgG in all genotypes including HbC and HbS alleles was confirmed by CSP and schizont extract, both considered markers of exposure (Table 2, Figure 2). However, significantly higher levels of total IgG against *P. falciparum* antigens were observed in the rural samples only against EBA-175 in the overall comparisons, and against AMA1 in the comparisons between HbAA versus HbAC, and HbAA versus HbCC (Table 2). Differences observed in the primary analysis were tested by logistic regression including Hb genotype, age categories (1/9, 10/max years), gender and parasite status confirming the role of adaptive genotypes for most of the tested antigens (Table 3). Finally, a non malaria antigen, tetanus toxoid, was tested revealing no differences in any of the subsets (data not shown).

**Table 2:** Total IgG means of log transformed OD values against several blood stage antigens (AMA1, EBA-175, MSP-1_19_, MSP-2, MSP-3), circumsporozoite protein (CSP), and parasite schizont extract (PSE) of *Plasmodium falciparum* in urban and rural sera of Burkina Faso.

Antigen	Prevalence	Pairwise comparisons				
	Urban samples (N = 266)	AA vs AC	AC vs CC	AA vs CC	AA vs AS	Overall
AMA1	78%	P = 0.001	NS	P = 0.01	P = 0.001	P = 0.0001
EBA-175	73%	P = 0.04	NS	P = 0.01	P = 0.03	P = 0.02
MSP-1_19_	67%	NS	P = 0.03	P = 0.01	P = 0.01	NS (P = 0.06)
MSP-2	64%	P = 0.001	NS	P = 0.03	P = 0.004	P = 0.0001
MSP-3	70%	NS	NS	NS	P = 0.02	NS
CSP	65%	P = 0.05	NS	P = 0.03	P = 0.001	P = 0.03
PSE	74%	P = 0.01	NS	P = 0.04	P = 0.01	P = 0.05
	Rural samples (N = 121)					
AMA1	90%	P = 0.03	NS	P = 0.03	NS	NS
EBA-175	76%	NS (P = 0.07)	NS	NS	NS (P = 0.06)	P = 0.05
MSP-1_19_	70%	NS	NS	NS	NS	NS
MSP-2	82%	NS	NS	NS	NS	NS
MSP-3	77%	NS	NS	NS	NS	NS
CSP	68%	NS	NS	NS	NS	NS
PSE	78%	NS	NS	NS	NS	NS

**Table 3:** Geometric means and 95% Confidence intervals in brackets of log transformed OD values against several blood stage antigens (AMA1, EBA-175, MSP-1_19_, MSP-2, MSP-3), circumsporozoite protein (CSP), and parasite schizont extract (PSE) of *Plasmodium falciparum* in urban and rural sera of Burkina Faso.

Antigen	AA	AC	AS	CC	SC	SS
	Urban Samples (N = 266)					
AMA1	2.90 (2.78–3.03)	3.14 (3.06–3.22)	3.20 (3.10–3.31)	3.27 (3.16–3.38)	3.23 (3.05–3.41)	3.40 (3.35–3.46)
EBA-175	2.75 (2.70–2.81)	2.82 (2.78–2.87)	2.84 (2.77–2.92)	2.91 (2.79–3.04)	2.82 (2.65–2.99)	3.15 (3.05–3.25)
MSP-1_19_	2.45 (2.34–2.58)	2.56 (2.46–2.66)	2.67 (2.54–2.81)	2.83 (2.60–3.08)	2.60 (2.38–2.85)	2.81 (2.58–3.05)
MSP-2	2.97 (2.84–3.10)	3.19 (3.13–3.26)	3.22 (3.12–3.32)	3.26 (3.15–3.38)	3.38 (3.36–3.40)	3.40 (3.35–3.46)
MSP-3	2.44 (2.35–2.53)	2.50 (2.43–2.58)	2.60 (2.50–2.71)	2.55 (2.37–2.74)	2.66 (2.38–2.97)	2.52 (2.45–2.61)
CSP	2.74 (2.69–2.80)	2.82 (2.77–2.87)	2.88 (2.82–2.95)	2.88 (2.75–3.01)	2.85 (2.70–3.01)	__
PSE	2.50 (2.44–2.57)	2.60 (2.56–2.65)	2.64 (2.56–2.72)	2.66 (2.55–2.77)	2.63 (2.46–2.82)	2.74 (1.26–3.95)
	Rural samples (N = 121)					
AMA1	3.12 (3.04–3.20)	3.27 (3.18–3.36)	3.09 (2.54–3.76)	3.31 (3.23–3.40)	3.06 (2.59–3.62)	3.39*
EBA-175	2.82 (2.74–2.90)	2.94 (2.85–3.03)	3.03 (2.76–3.34)	2.95 (2.81–3.08)	2.70 (2.28–3.20)	3.16*
MSP-1_19_	2.82 (2.60–3.06)	2.54 (1.31–4.92)	1.92*	__	__	__
MSP-2	2.82 (2.70–2.94)	2.87 (2.75–2.99)	2.82 (2.37–3.35)	2.89 (2.57–3.25)	2.88 (2.41–3.45)	3.24*
MSP-3	2.57 (2.48–2.67)	2.71 (2.58–2.84)	2.52 (2.11–3.01)	2.69 (2.41–3.01)	2.86 (1.03–5.97)	2.55*
CSP	2.80 (2.75–2.85)	2.82 (2.77–2.86)	2.88 (2.82–2.94)	2.76 (2.73–2.79)	2.77 (2.68–2.85)	__
PSE	2.55 (2.46–2.65)	2.61 (2.49–2.74)	2.72 (2.55–2.90)	2.59 (2.48–2.70)	2.56 (0.83–7.89)	2.62*

* single individual

## Discussion

Despite substantial evidence of protection against clinical malaria given by the haemoglobin variants HbC and HbS, the precise mechanism(s) are still under debate. A recently proposed mechanism suggested that the protection conferred by HbC may be attributed to the reduction of cytoadherence and impaired rosetting observed in relation to the altered display of PfEMP1 on HbCC erythrocytes [7], [8] which raises the question of whether this would affect VSA recognition, and ultimately the immune reactivity against VSA. Taking on from this hypothesis we anticipated a difference in antibody levels of HbCC homozygous, possibly a reduction due to their impaired VSA expression. Also, an “immunological hypothesis” initially proposed for HbS has been supported by a number of studies in various epidemiological contexts although the functional mechanisms involved in the enhanced acquisition of natural immunity observed in HbAS remain elusive [9], [10]. Therefore, we investigated haemoglobin phenotype-specific immune reactivity to a composite isolate expressing a wide range of *P. falciparum* VSAs in subjects resident in two different malaria endemic contexts of Burkina Faso. Results showed that the reported abnormal cell-surface display of *Pf*EMP1 on HbC infected erythrocytes observed *in vitro* is not associated to lower anti- *Pf*EMP1 humoral response *in vivo*. In fact, higher immune response against the *P. falciparum* VSA panel and several antigens were observed in all adaptive genotypes containing at least one allelic variant HbC or HbS in the low transmission urban area (Figure 1). Interestingly, cross-reactive antibody responses, i.e. those directed against erythrocyte surface expressed parasite antigens from heterologous parasite isolates are an important component of acquired immunity against malaria [17]–[19]. However, no differences were detected in the high transmission rural areas. Significantly higher levels of antibodies at several *P. falciparum* antigens were also observed in the same urban samples (Table 2, Table 3, Figure 2). These differences were not explained by age or parasite status at that time point as confounders for most of the antigens examined (Table 4), rather reflected the cumulative effect of a life long exposure to *P. falciparum* malaria in the presence of a protective factor such as HbC and/or HbS. In both contexts the response against tetanus toxoid was not influenced by the β-globin genotype. Thus, these findings suggest that both HbC and HbS could promote the early development of naturally acquired immunity against malaria. The enhanced immune reactivity in both HbC and HbS carriers supports the hypothesis that the protection against malaria of these adaptive genotypes might be at least partially mediated by acquired immunity against malaria.

**Table 4:** Logistic regression analysis of antibody levels against all *Plasmodium falciparum* tested antigens

Antigen	Variable			
	Hb genotype	Age	Gender	Parasite +
AMA1	P = 0.001	NS	NS	NS
EBA-175	NS	NS	NS	NS
MSP-1_19_	NS (P = 0.06)	NS	NS	NS
MSP-2	P = 0.003	NS	NS	NS
MSP-3	NS (P = 0.08)	NS	NS	NS
CSP	P = 0.004	NS	NS	NS
PSE	P = 0.01	P = 0.01	NS	NS
A4 MFI	NS	NS	NS	NS
CI MFI	P = 0.05	NS	NS	NS

We believe that the discrepancy between results from urban and rural settings could be the consequence of saturated immunity in high transmission contexts. These observations emphasize the need, when studying candidates of genetic resistance/susceptibility to malaria and their underlying hypothesized mechanisms, to take into full account the epidemiological context as a potential confounder.

Although this study can not conclusively validate any of the different functional mechanisms proposed to explain the protection of haemoglobin variants, a consistently enhanced immune reactivity in both HbC and HbS adaptive genotypes suggests the idea of a convergence in terms of their impact on the acquisition of immunity against malaria. It has been already shown that the model initially proposed for explaining G6PD protection, by enhanced phagocytosis of ring- parasitized altered erythrocytes, fits well also in the case of HbS and β-thalassemia [20]. Thus, it may be possible that this mechanism may also be extended to the case of HbC. Further investigation in the rural areas so far examined, focusing on young children, especially below five years, is undergoing to unravel the modulation exerted by HbC (and HbS) on the onset of naturally acquired immunity to malaria.
